# Editorial: Microbial associations formed and hosted by protists, algae, and fungi

**DOI:** 10.3389/fmicb.2023.1341058

**Published:** 2024-01-05

**Authors:** Alexey Potekhin, Andrey O. Plotnikov, Natalia Gogoleva, Bettina Sonntag

**Affiliations:** ^1^Research Department for Limnology, Mondsee, University of Innsbruck, Mondsee, Austria; ^2^Institute for Cellular and Intracellular Symbiosis of the UrB RAS, Orenburg Federal Research Center, Orenburg, Russia; ^3^Laboratory of Environmental Metagenomics, Institute of Fundamental Medicine and Biology, Kazan Federal University, Kazan, Russia

**Keywords:** microbiome, protists, algae, microbial communities, 16S rRNA gene metabarcoding, microbiome function, symbiosis, prokaryotic symbionts

Microbial associations hosted by protists, algae, and fungi remain out of the research mainstream. Prokaryotic and eukaryotic microorganisms share the same environments, form complex communities, and are inseparable in nature. However, there is an artificial gap in human knowledge between microbiology and protistology, which undoubtedly will be closed in the future due to emerging interest in microbial communities. The microbiomes of unicellular eukaryotes probably play an important role in the environmental adaptation and survival of both hosts and associates, a role that still has to be elucidated. The surface of multicellular algae provides generous substrates for different microorganisms, whose functional role in host biology needs to be figured out. This Research Topic provides new insights into different aspects of the structure and functions of the microbiomes associated with protists and algae.

Some members of the microbiomes of protists can be professional symbionts (Lanzoni et al., 2019; Husnik et al., 2021), but the majority of the prokaryotic associates of protists are, likely, occasional (Plotnikov et al., 2019; Zhang et al., 2023), and, thus, should be considered as provisional symbionts. Giddings et al. recorded for the first time slime mold *Stemonitis* sp. in acid rock drainage. Metagenomic sequencing and FISH showed that these amoebae hosted *Dyella terrae* (Xanthomonadales), which is known to be an acid-tolerant and metal-resistant free-living bacterium. The bacteria were also present outside of the host, the non-reduced bacterial genome also indirectly witnessed that the symbiosis was facultative. The bacteria had type IV secretion system genes that could aid in their ability to colonize amoeba cells and to resist *Stemonitis* predation. The bacteria could find shelter inside amoeba cells, while the host could benefit either from metabolites and nutrients provided by the occasional symbionts in the poor extreme environment or simply maintain them as a “symbiotic farm” and consume upon starvation.

The other protist that is known to settle a “food farm” on the cell surface is a ciliate *Kenthrophoros flavus*. This astomatous ciliate possesses thiotrophic ectosymbionts (“*Ca*. Kentron”, Gammaproteobacteria) that are used as a food source by the ciliate (Seah et al., 2017). By PacBio sequencing of the cloned 16S rRNA genes, Bi et al. detected a major symbiont of *Kenthrophoros*, a new species of “Ca. Kentron”, and another bacterium from the family *Muribaculaceae* (Bacteroidales), whose members are known from mouse and human gut microbiomes (Lagkouvardos et al., 2019). This bacterium called MLAKF was present stably and in big numbers on the surface of the ciliate host cells together with “Ca. Kentron”. The authors hypothesize a tripartite symbiosis, suggesting that MLAKF could supply “*Ca*. Kentron” ectobionts limited in their ability to fix CO_2_ with extra organic carbon produced by degrading carbohydrates, and both bacteria could be used by the ciliate as a preferential food under diverse conditions.

Sometimes the unicellular hosts' fitness depends on their microbiomes. Himi et al. demonstrated that *Paramecium bursaria*, a ciliate that maintains unicellular green algae in its cytoplasm and is able to survive consuming only the sugars produced by the photosynthetic symbionts, harbors a taxonomically rich bacterial community. The host fitness decreased significantly under non-lethal treatment with antibiotics, confirming that the microbiomes were essential for the ciliates. The sublines of the *P. bursaria* strain that was for several years maintained in the absence of exogenous bacterial food were examined by the 16S rRNA gene metabarcoding. Interestingly, the sets of associated bacteria were quite different among well- and poorly proliferating lines, while the lines of the same proliferation mode manifested a highly similar composition of their bacterial communities. Thus, the disrupted microbiome of ciliates could be the reason for differences in their fitness.

Le Reun et al. investigated the microbiome associated with the marine diatom *Actinocyclus* sp. The authors used co-cultivation to examine the growth-promoting capabilities of 15 bacterial isolates of different taxa from the bacterial community associated with this diatom. The diatoms were shown to grow differently in association with this or that particular member of their microbiome. The diatom growth was enhanced by several bacterial strains from the associated community, but the beneficial effects of different associates were observed in different time periods. Probably, the dynamics of the microbiome followed the changes in the pool of chemicals released by the diatom, similar to the dynamics of microbial community during phytoplankton blooms (Jung et al., 2021).

Currently not only the complete genomes of all partners in symbiotic association can be obtained and assembled from one metagenomic dataset, but the metabolic abilities of the microbial community can also be predicted. Wang et al. described that differences in the predicted functional profiles of the epiphytic bacterial communities on the surface of the marine macroalga *Sargassum thunbergii* male and female specimens and their receptacles were accompanied by differences in the structure of the microbiomes. Representatives of numerous bacterial taxa formed a core microbiome of *S. thunbergii* as they were always found in all analyzed microbiomes. Probably, it is mainly the nutrient cycling between algae and bacteria that shapes this core community and serves as a basis for a long and stable relationship between algae and bacteria (Christie-Oleza et al., 2017). However, the physiology of the communities associated with male and female specimens and their receptacles was different. Some specific associates exploited metabolic differences of the colonized hosts and their organs, responding positively to the phenolic compounds that are more abundant in the tissues of the female algae. Thus, the sex of the host alga influences the microbiome. Not all of the microbiome members were beneficial for the host, and the functions of bacteria with low abundances might also be important in the community, adding complexity to the relations between the host and its microbiome.

All articles included in this Research Topic provide some evidence that the microbiomes of protists and algae play significant roles in the biology of their hosts. These roles include, but are not limited to, being a food source for the host, providing metabolic aid and compensation, host protection, and community maintenance. The articles clearly point the perspective for methods of protist and algae microbiomes management to enhance the harvest of hosts in laboratories and biotechnology plants.

## Author contributions

AP: Writing—original draft. AOP: Writing—review & editing. NG: Writing—review & editing. BS: Writing—review & editing.
